# Supplementary material for: A habenula-insular circuit encodes the willingness to act
Source: Nat Commun. 2021 Nov 3;12:6329. doi: 10.1038/s41467-021-26569-1 (PMC8566457; doi:10.1038/s41467-021-26569-1)
Supplement: Supplementary file 1 — Supplementary Information [file 41467_2021_26569_MOESM1_ESM.pdf]

A habenula-insular circuit encodes the willingness to act

Nima Khalighinejad[1,3*], Neil Garrett[1,3], Luke Priestley[1,3], Patricia Lockwood[1,2], Matthew FS Rushworth[1]

Affiliations

[1]Wellcome Centre for Integrative Neuroimaging, Department of Experimental Psychology, University of Oxford, Oxford OX1 3SR, UK

[2]Centre for Human Brain Health, School of Psychology, University of Birmingham, Birmingham, UK

[3]Equal contribution

*** To whom correspondence should be addressed:**

Address: Nima Khalighinejad,
Wellcome Centre for Integrative Neuroimaging,
Department of Experimental Psychology,
University of Oxford,
Oxford, UK

E-mail: nima.khalighinejad@psy.ox.ac.uk

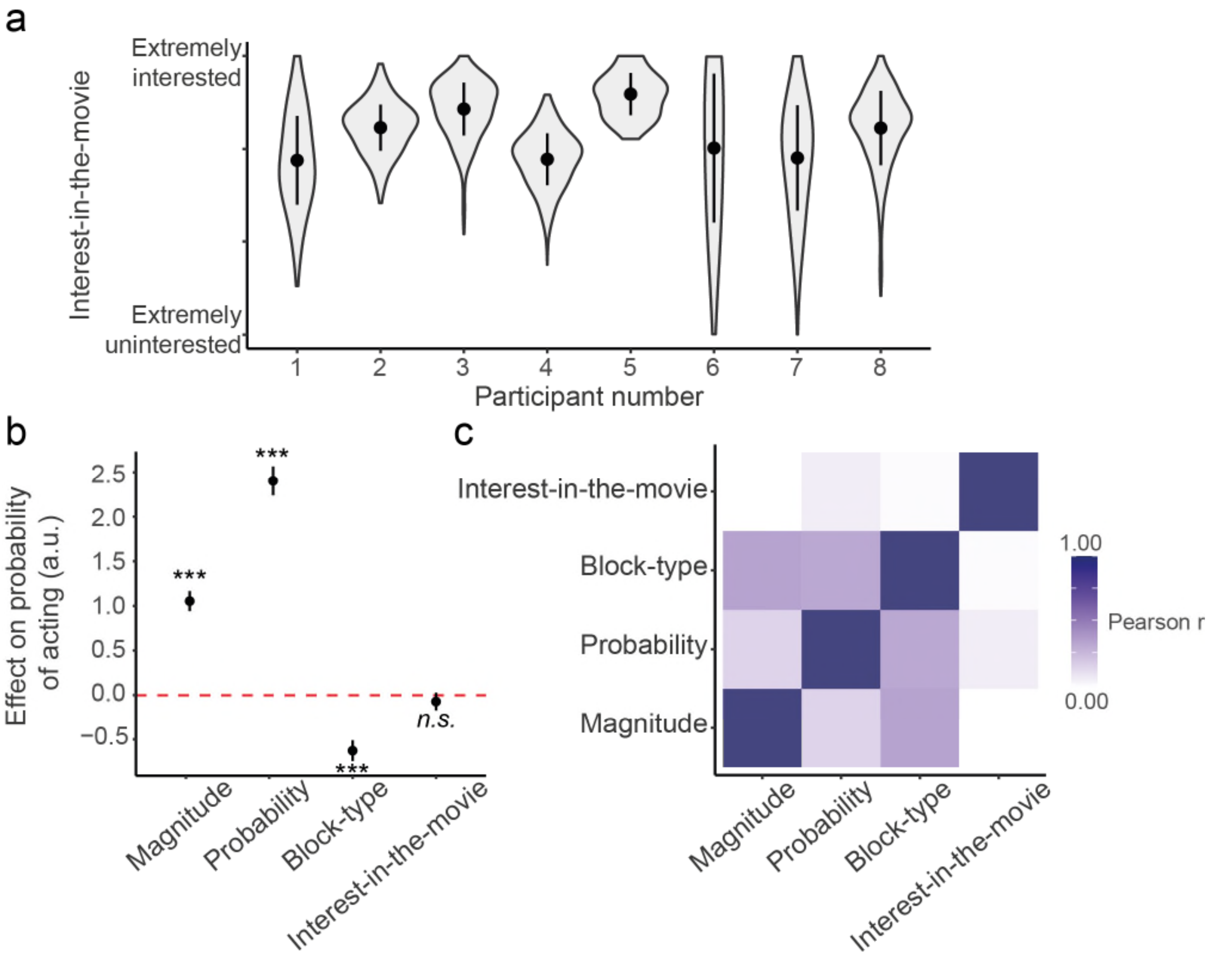

Figure S1. Related to Figure 1. We performed a control behavioural experiment to show that interest in the movie scenes does not influence participants' decision about whether or not to act. Participants (n=8) performed two versions of the task, on different days. One version was exactly identical to the original design: participants were presented with offers on the screen and had to decide whether to accept an offer and engage in an effortful task for potential reward. In the second version of the task participants were asked to rate their interest in the movie clip: at each trial, rather than being presented with offers, they were asked to rate their momentary interest in the movie on the scale of 1-9 (1=extremely uninterested; 9=extremely interested). They responded by pressing the number corresponding to their interest-rating on the keyboard. Importantly, for each participant, the timing of the interest-in-the-movie rating question and the offer presentation exactly matched between the two versions of the task. The order of the experiments was counterbalanced between the participants. Half performed the original version followed by the rating task and the other half in reverse order. (a) interest-in the-movie-clip rating varied from trial-to-trial and from subject-to-subject, to different extents. In the violin plots the black circles are the mean and the whiskers are the standard deviation. (b) Participants were more likely to act when offered higher magnitude rewards (β=1.05±0.11, P=2e-16), higher probability rewards

(β=2.39±0.16, P=2e-16), and when they were in a poor compared to a rich block (β=-0.63±0.12, P=6.56e-08), replicating the main behavioural findings from the main experiment. interest-in-the-movie-clip, however, had no significant effect on probability of acting (β=-0.07±0.10, P=0.463), nor was it correlated with other contextual factors in participants' environments including reward magnitude, reward probability, and block type (c). In (b), the dots show the mean standardized coefficients from the linear mixed-effect model and whiskers are standard error of the mean of the estimated coefficients. Generalised linear mixed-effect model with statistical significance assessed via asymptotic two-sided Wald test as implemented in *lme4*. *** P<0.001, n.s. not significant. a.u. arbitrary units. Source data are provided as a Source Data file.

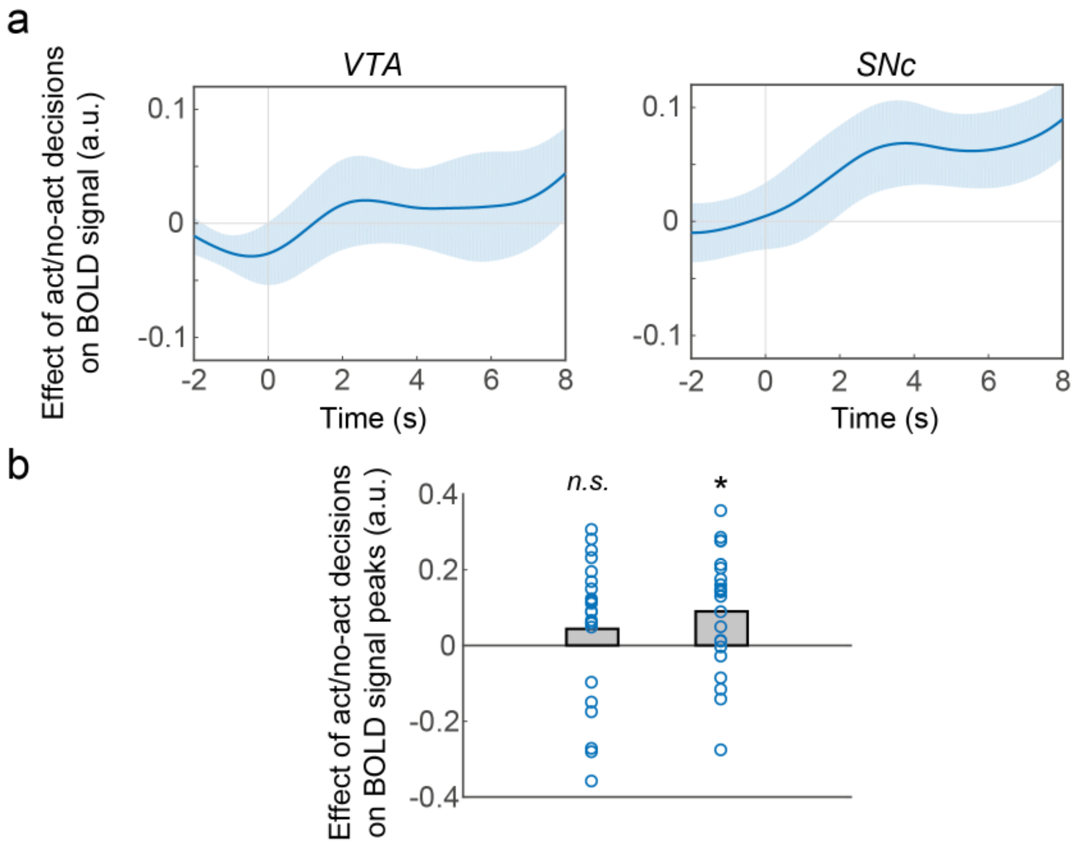

Figure S2. Related to Figure 2. We found a negative relationship between act/no-act decisions and BOLD activity in MidD (see Fig.2a). This might be counterintuitive given that others[18] and our own findings[16] have previously showed that MidD encodes action initiation. Accordingly, one would expect MidD to positively encode the response effect. One way to address this discrepancy directly is simply to separate the subdivisions of MidD (substantia nigra pars compacta (SNc) and ventral tegmental area (VTA) based on the Pauli Atlas[24]; see Methods) and investigate the effect of response (act/no-act decisions) on BOLD signals time locked to action onset (rather than stimulus onset as in Figure 2). We found – in accordance with previous reports –a positive deflection in SNc when time-locking to action onset (a,b) (P=0.015; GLM2.1). The positive SNc activity – when time locked to action onset – corresponds with the role of SNc in action initiation, once the haemodynamic lag is taken into account. This shows that from the negative activity pattern that is time-locked at stimulus onset and which is seen when averaging activity across MidD (Fig.2), one cannot conclude that nowhere in MidD encodes action initiation; the SNc division of MidD encodes action initiation but it does so at the time of action. The lines and shadings show the mean and standard error of the β weights across the participants, respectively. Time zero is the action onset. In (b) each ring represents one participant (n=22). Significance testing on time-course data was performed by using a leave-one-out procedure on the group peak signal. Two-sided, one-sample t-tests. * P<0.05. n.s. not significant. a.u. arbitrary units.

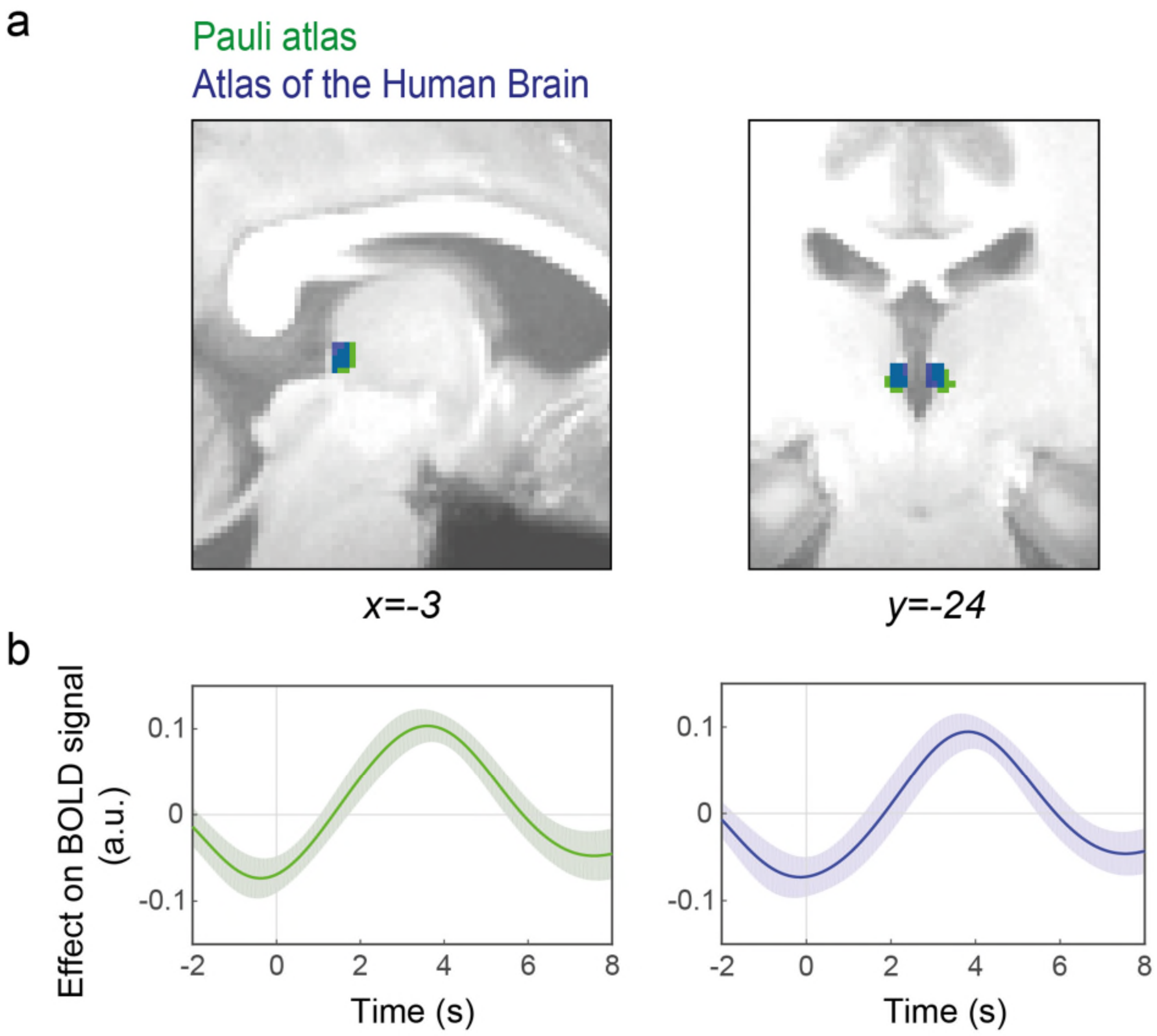

Figure S3. Related to Figure 3. To facilitate between-study comparisons, we designed the HB mask using the Atlas of the Human Brain[49], similar to our previous study[16]. However, to assess the robustness of the effect of willingness-to-act on HB BOLD signal, we tried to replicate the same effect using a different HB mask from an independent subcortical atlas[24]. (a) The original HB mask (in blue) is shown overlaid over the Pauli and colleagues (2017) mask (in green). (b) The effect of willingness-to-act on BOLD signal extracted from the blue mask is similar to that extracted from the green mask. The lines and shadings show the mean and standard error of the β weights across the participants, respectively. Time zero is the trial onset. a.u. arbitrary units.

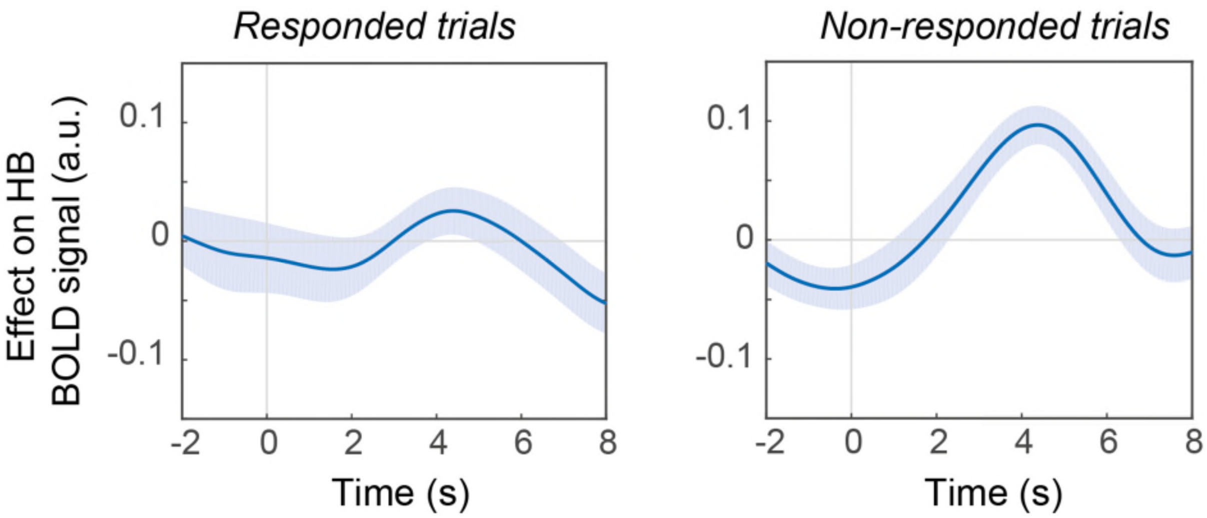

Figure S4. Related to Figure 3. The effect of willingness-to-act on habenula (HB) BOLD signal plotted separately for trials in which participants decided to make an action (response), and those in which they withheld an action (refrained from responding). The stronger relationship between HB activity and willingness-to-act in non-responded trials could be simply due to the fact that the variation of willingness-to-act was different for trials in which participants acted compared to trials in which they did not act. To test this hypothesis, we compared the variation of willingness-to-act between the act and not-act trials. The mean variation across participants in willingness-to-act for action trials was 0.23 (±0.05). It was 0.21 (±0.04) for trials in which participants did not act. Importantly, the difference between the two was not statistically significant: Two-sided, paired-samples t-test; p=0.12. This suggests that the stronger relationship between HB and willingness-to-act in non-responded trials could not be simply explained by the difference in variation in willingness-to-act. The lines and shadings show the mean and standard error of the β weights across the participants, respectively. Time zero is the trial onset. a.u. arbitrary units.

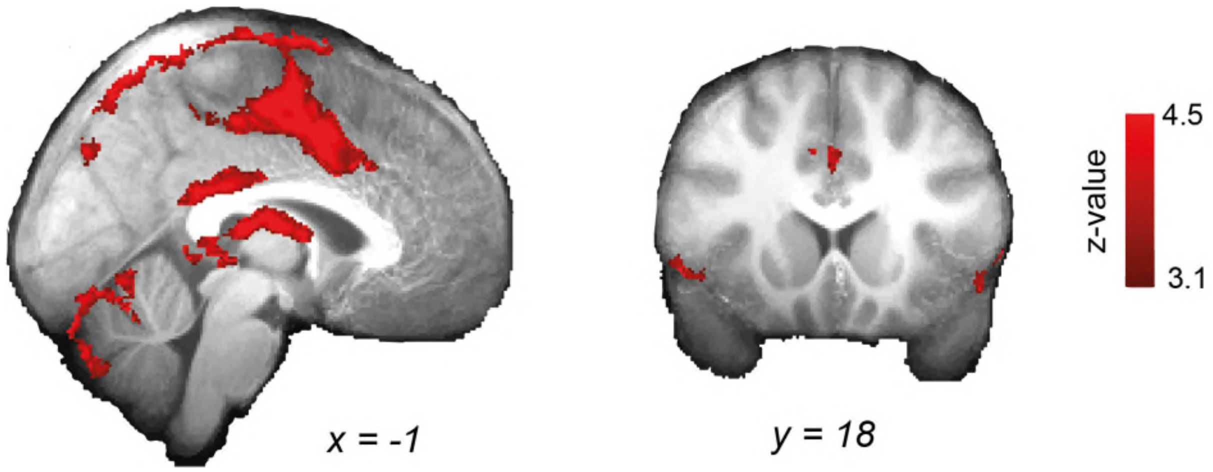

Figure S5. Related to Figure 4. Whole-brain analysis showing voxels where activity reflected the response contrast (act/no-act decisions; Methods; GLM1). Whole-brain cluster-based correction, Z>3.1, overlaid on averaged structural image of all subjects in standard space.

Predictor	Beta	SE	p	Δ chi-sq
totalTime	-0.74954	0.14182	1.26E-07	27.9318
reward	2.07133	0.26356	3.87E-15	48.6404
probability	3.52693	0.26038	2E-16	167.4648
block	-0.85856	0.18014	1.88E-06	16.3345
expectedValue(t-1)	-0.15845	0.08599	.065	3.3951
response(t-1)	-0.06599	0.17124	.699	0.1485
*reward*probability*	1.25198	0.21718	8.18E-09	33.2325
*reward*block*	-0.64485	0.17907	0.000317	12.9672
*probability*block*	-0.69612	0.20548	0.000704	11.4774

Table S1. Related to Figure 1. Regression coefficients (Beta) from the generalised linear mixed-effect model used to predict participants' action decisions at each trial. SE is standard error. Generalised linear mixed-effect model with statistical significance assessed via asymptotic two-sided Wald test as implemented in *lme4*. Source data are provided as a Source Data file.

Bayesian One Sample T-Test

ROI	BF_{10}	error %
DS	0.562	0.019
NAc	0.223	0.031
MidD	0.330	0.030
PPN	1.064	0.005
HB	91.238	6.304e -5
BF	2.312	4.602e -4

Table S2. Related to Figure 3. There is very strong evidence in support of our hypothesis in habenula (HB) (Bayes factor (BF_{10})>30) but no evidence in other areas (BF_{10}<1). There is anecdotal evidence in basal forebrain (BF), but it is evident in Figure 3 that the positive deflection in BF happens before stimulus presentation and therefore could not be related to encoding of willingness-to-act. Bayesian two-sided, one-sample, t-test was performed in JASP using the default prior. DS (dorsal striatum); NAc (nucleus accumbens); MidD (midbrain dopaminergic system); PPN (pedunculopontine nucleus).

Willingness-to-act contrast						
**Cluster**	**No. of voxels**	**P value**	**Z-max**	**X (mm)**	**Y (mm)**	**Z (mm)**
Postcentral gyrus (left)	9327	3.64E-44	6.11	-48	-23	56
Cerebellum	6691	4.15E-35	5.66	18	-51	-24
Anterior insula (right)	3366	1.1E-21	5.16	43	16	1
Supplementary motor area	3285	2.59E-21	5.50	-6	-2	54
Anterior insula (left)	893	5.96E-08	5.06	-32	20	5
Supramarginal gyrus (right)	631	3.81E-06	4.19	44	-37	54
Postcentral gyrus (right)	617	4.89E-06	4.83	52	-24	49
Thalamus (left)	369	0.0007	4.35	-15	-25	10

Table S3. Related to Figure 4. Full list of clusters related to willingness-to-act contrast. Here we focussed on the anterior insula and supplementary motor area. Harvard-Oxford Cortical and Subcortical Structural Atlas was used for labelling.

Path	***β***	***SE***	***p***
Anterior insula → Habenula	0.158	0.006	<.001
Basal forebrain → Midbrain dopamine	0.040	0.005	<.001
Pedunculopontine nucleus → Midbrain dopamine	0.632	0.005	<.001
Habenula → Midbrain dopamine	-0.010	0.005	0.037
Midbrain dopamine → Dorsal striatum → Nucleus accumbens	-0.021<br>0.162	0.006<br>0.005	<.001<br><.001
Dorsal striatum → SMA	0.305	0.006	<.001
Nucleus accumbens → SMA	-0.174	0.006	<.001

Table S4. Related to Figure 6. Estimates of the path coefficients (β) of structural equation modelling (SEM). The arrows show the direction of the influence. SE is standard error. Structural equation model with statistical significance determined via two-sided Wald test as implemented in *lavaan*.
