# Supplementary material for: A habenula-insular circuit encodes the willingness to act
Source: Nat Commun. 2021 Nov 3;12:6329. doi: 10.1038/s41467-021-26569-1 (PMC8566457; doi:10.1038/s41467-021-26569-1)
Supplement: Supplementary file 3 — Reporting Summary [file 41467_2021_26569_MOESM3_ESM.pdf]

nature research

Corresponding author(s): Nima Khalighinejad

Last updated by author(s): Sep 27, 2021

# Reporting Summary

Nature Research wishes to improve the reproducibility of the work that we publish. This form provides structure for consistency and transparency in reporting. For further information on Nature Research policies, see our Editorial Policies and the Editorial Policy Checklist.

## Statistics

For all statistical analyses, confirm that the following items are present in the figure legend, table legend, main text, or Methods section.

| n/a | Confirmed | |
|---|---|---|
| ☐ | ☒ | The exact sample size (*n*) for each experimental group/condition, given as a discrete number and unit of measurement |
| ☐ | ☒ | A statement on whether measurements were taken from distinct samples or whether the same sample was measured repeatedly |
| ☐ | ☒ | The statistical test(s) used AND whether they are one- or two-sided<br>*Only common tests should be described solely by name; describe more complex techniques in the Methods section.* |
| ☐ | ☒ | A description of all covariates tested |
| ☐ | ☒ | A description of any assumptions or corrections, such as tests of normality and adjustment for multiple comparisons |
| ☐ | ☒ | A full description of the statistical parameters including central tendency (e.g. means) or other basic estimates (e.g. regression coefficient) AND variation (e.g. standard deviation) or associated estimates of uncertainty (e.g. confidence intervals) |
| ☐ | ☒ | For null hypothesis testing, the test statistic (e.g. *F*, *t*, *r*) with confidence intervals, effect sizes, degrees of freedom and *P* value noted<br>*Give P values as exact values whenever suitable.* |
| ☐ | ☒ | For Bayesian analysis, information on the choice of priors and Markov chain Monte Carlo settings |
| ☐ | ☒ | For hierarchical and complex designs, identification of the appropriate level for tests and full reporting of outcomes |
| ☐ | ☒ | Estimates of effect sizes (e.g. Cohen's *d*, Pearson's *r*), indicating how they were calculated |

*Our web collection on statistics for biologists contains articles on many of the points above.*

## Software and code

Policy information about availability of computer code

| | |
|---|---|
| Data collection | The experiment was written in Matlab R2017 (Mathworks, Natick, USA), using the Psychophysics Toolbox extension |
| Data analysis | The data analysis was performed in Matlab R2017a (Mathworks, Natick, USA) and R version 3.4.1. Structural equation modelling was conducted with Latent Variable Analysis (lavaan) package v.0.6–4 in R. The mixed-effect modelling was performed with the 'lme4' and 'optimx' packages in R. Pre-processing of fMRI data was performed using tools from FMRIB Software Library (FSL).<br>Custom written R scripts used for measuring "willingness-to-act" and reproducing the figures related to behavioural analysis are available at: https://doi.org/10.5061/dryad.6t1g1jwxq |

For manuscripts utilizing custom algorithms or software that are central to the research but not yet described in published literature, software must be made available to editors and reviewers. We strongly encourage code deposition in a community repository (e.g. GitHub). See the Nature Research guidelines for submitting code & software for further information.

## Data

Policy information about availability of data

All manuscripts must include a data availability statement. This statement should provide the following information, where applicable:

- Accession codes, unique identifiers, or web links for publicly available datasets
- A list of figures that have associated raw data
- A description of any restrictions on data availability

Data availability
Data files and materials used in the main analyses presented here (Fig1c-f; Fig.2; Fig.3; Fig.4b-d; Fig.5) have been archived and uploaded to the Data DRYAD and are freely available at: https://doi.org/10.5061/dryad.6t1g1jwxq
Source data are provided with this paper.

Code availability
Custom written R scripts used for measuring “willingness-to-act” and reproducing the figures related to behavioural analysis are available at: https://doi.org/10.5061/dryad.6t1g1jwxq

# Field-specific reporting

Please select the one below that is the best fit for your research. If you are not sure, read the appropriate sections before making your selection.

☐ Life sciences ☒ Behavioural & social sciences ☐ Ecological, evolutionary & environmental sciences

For a reference copy of the document with all sections, see nature.com/documents/nr-reporting-summary-flat.pdf

# Behavioural & social sciences study design

All studies must disclose on these points even when the disclosure is negative.

| | |
|---|---|
| Study description | Data are quantitative experimental data. |
| Research sample | 25 participants (18 females), aged 18-40 years participated in the study. Participants included students, university staff, and public members from Oxford. The participants were, therefore, representative of a university town in the UK. Note that all analyses focused on behavioral and neural data collected from all participants and there was no attempt to define or to compare different sub-groups of participants. |
| Sampling strategy | Random sampling was used. The sample size was based on a previous ultra-high field neuroimaging study which used a comparable experimental paradigm (Khalighinejad et al., PNAS, 2020). |
| Data collection | Participants practiced the task outside the MRI room where force calibration was also measured. Force data was recorded using a TSD121B-MRI dynamometer (BIOPAC Systems Inc., USA) running on an MP160 acquisition device (BIOPAC Systems Inc., USA). The scanning was then performed after training in the MRI room (Siemens 7T MRI scanner) adjacent to another room where the researcher and the radiographer were present. The researcher was not blinded to experimental condition nor the study hypothesis. |
| Timing | Data collection took place during spring and summer 2019. |
| Data exclusions | One subject was excluded from all behavioural and brain analyses for failing to respond frequently enough according to an a priori exclusion criterion (response rate of more than 85% or less than 15%). In addition, two subjects were excluded from all brain analyses due to excessive head motion (absolute mean displacement > 2mm; a priori estabilished). |
| Non-participation | All participants completed the study. |
| Randomization | The study had a within-subject design and therefore no group allocation was necessary. |

# Reporting for specific materials, systems and methods

We require information from authors about some types of materials, experimental systems and methods used in many studies. Here, indicate whether each material, system or method listed is relevant to your study. If you are not sure if a list item applies to your research, read the appropriate section before selecting a response.

## Materials & experimental systems

| n/a | Involved in the study | |
|---|---|---|
| ☒ | ☐ | Antibodies |
| ☒ | ☐ | Eukaryotic cell lines |
| ☒ | ☐ | Palaeontology and archaeology |
| ☒ | ☐ | Animals and other organisms |
| ☐ | ☒ | Human research participants |
| ☒ | ☐ | Clinical data |
| ☒ | ☐ | Dual use research of concern |

## Methods

| n/a | Involved in the study | |
|---|---|---|
| ☒ | ☐ | ChIP-seq |
| ☒ | ☐ | Flow cytometry |
| ☐ | ☒ | MRI-based neuroimaging |

## Human research participants

Policy information about studies involving human research participants

| | |
|---|---|
| Population characteristics | See above. |
| Recruitment | Participants were recruited through email advertisements, poster boards, and departmental database. Psychology/neuroscience students are more likely to have self-selected to participate in the study. However, due to the parametric |

nature of our within-participant design and analysis approach, we believe that this is unlikely to have influenced the results.

Ethics oversight

Ethical approval was given by the Oxford University Central University Research Ethics Committee (CUREC) (Ref-Number: MSD-IDREC-R55856/RE001).

Note that full information on the approval of the study protocol must also be provided in the manuscript.

# Magnetic resonance imaging

## Experimental design

Design type

Event-related

Design specifications

The task included a total of 216 trials, which were divided into 6 blocks of 36 offers (trials). While watching a movie, participants received a series of offers in the form of visual stimuli that were superimposed upon the movie. Each offer appeared for 2s and could be accepted by button-pad response while it remained on-screen. If the participant made a response to accept an offer, they needed to complete a short effort-task for a duration of 3-4s. Depending on the performance, feedback about the reward outcome was superimposed on-screen for 2s. The next offer appeared after an inter-trial-interval (ITI) of 4-5s.

Behavioral performance measures

All button presses and response times were recorded. To model the behaviour we used a generalised linear mixed-effect model with by-subject random intercept and by-subject random slopes. The modelling was performed with the 'lme4' and 'optimx' packages in R. Subjects were expected to have a response rate between 15% and 85%. One subject was excluded from the analysis for not meeting this criteria.

## Acquisition

Imaging type(s)

Functional and structural

Field strength

7T

Sequence & imaging parameters

Structural and functional MRI was collected using a Siemens 7T MRI scanner. High-resolution functional data was acquired using a multiband gradient-echo T2* echo planar imaging (EPI) sequence with a 1.5 x 1.5 x 1.5 mm resolution; multiband acceleration factor 3; repetition time (TR) 1962 ms; echo time (TE) 20 ms; flip angle 66 degrees; and a GRAPPA acceleration factor 2. Field of view (FOV) was adjusted to cover the whole-brain with axial orientation and a fixed angulation of -30 degrees (anterior-to-posterior phase encoding direction; 96 slices). Additionally, a single-measurement, whole-brain, functional image was acquired prior to the main functional image (with similar orientation). This 'pre-saturation' scan was later used to improve registration of the main functional image to the whole brain. Structural data was acquired with a T1-weighted MP-RAGE sequence with a 0.7 x 0.7 x 0.7 mm resolution; GRAPPA acceleration factor 2; TR 2200 ms; TE 3.02 ms; and inversion time (TI) 1050 ms. To correct for field inhomogeneities a separate Fieldmap sequence was acquired with a 2 x 2 x 2 mm resolution; TR 620 ms; TE1 4.08 ms; TE2 5.10 ms. To regress out the effect of physiological noise in functional data, cardiac and respiratory frequencies were collected by pulse oximetry and respiratory bellows.

Area of acquisition

Whole brain

Diffusion MRI ☐ Used ☒ Not used

## Preprocessing

Preprocessing software

Pre-processing was performed using tools from FMRIB Software Library (FSL) (Jenkinson et al., 2012). Functional images were first normalised, spatially smoothed (Gaussian kernel with 3mm full-width half-maximum), and temporally high-pass filtered (3 dB cut-off of 100 s). The effect of participants' head motion during the scanning was removed by MCFLIRT (Jenkinson et al., 2002). The Brain Extraction Tool (BET) (Smith, 2002) was used on functional and structural images to separate brain from non-brain matter.

Normalization

The registration of functional images into Montreal Neurological Institute (MNI)-space was performed in three stages: (1) Whole-brain task EPI to pre-saturation EPI using FMRIB's Linear Image Registration Tool (Jenkinson & Smith, 2001) with 3 degrees of freedom (translation only). (2) Whole-brain EPI to individual structural image using Boundary-Based Registration (BBR) (Greve & Fischl 2009) by incorporating Fieldmap correction. (3) Individual structural image to Standard image by using FMRIB's Non-linear Image Registration Tool (FNIRT).

Normalization template

The MNI152 template was used.

Noise and artifact removal

To model instant signal distortions due to changes in the magnetic field caused by performing the effort-task we added two constant regressors that were not convoluted with HRF (nonConv). These regressors started at the beginning of the TR when the response was recorded and the effort-task started (nonConvResp), and when the outcome phase started (nonConvOut). They had a duration of one TR (1.96 s). To further reduce variance and noise in the BOLD signal, we also added task-unrelated confounds which included: (1) head motion parameters as estimated by MCFLIRT in the pre-processing stage; (2) voxelwise regressors created by physiological noise modelling (PNM) (Brooks et al., 2008) to model the effects of physiological noise (cardiac and respiratory); (3) regressors to completely remove timepoints with large motions that could not be fixed with linear methods.

Volume censoring

The FSL Motion Outlier tool was used to detect timepoints that have been corrupted by large motion. It created a confound matrix that were used in the GLM to completely remove the effects of these timepoints on the analysis, without any adverse affects in the statistics. This is intended to deal with the effects of intermediate to large motions, which corrupt images beyond anything that the linear motion parameter regression methods can fix. Using this method, on average, 5% (across participants std 3%) of the volumes were marked as corrupted by large motion.

## Statistical modeling & inference

Model type and settings

Whole-brain statistical analyses was performed at two-levels as implemented in FSL FEAT (Woolrich et al,. 2001). At the first level, we used a univariate general linear model (GLM) framework for each participant to compute the parameter estimates. The contrast of parameter estimates and variance estimates from each scanning session were then combined in a second-level mixed-effects analysis (FLAME 1+2), treating scanning sessions as random effect.
For time-series analyses, the filtered time-series of each voxel within each ROI was averaged, normalised and up-sampled (20 times). The up-sampled data was then interpolated using the cubic spline method and was epoched in 10 s windows, starting from 2 s before to 8 s after the trial onset (appearance of the offer). Time-series GLMs were then fit at each time step of the epoched data, using ordinary least squares (OLS).

Effect(s) tested

All linear models used to test the effects have been described in the Methods section. For whole brain analysis see GLM.1. For ROI-based analysis see GLM2.1, GLM2.2, GLM2.3, and GLM2.4.
Significance testing on time-course data was performed by using a leave-one-out procedure on the group peak signal to avoid potential temporal selection biases. For every participant, we estimated the peak signal time by identifying the peak in the time course of the mean beta weights of the relevant regressor in all other participants. When we did this, we identified the peak (positive or negative) of the regressor of interest within the full width of the epoched time course: from 2 s before to 8 s after the trial onset. Next, we took the beta weight of the remaining participant at the time of the group peak. We repeated this for all participants. Therefore, the resulting 22 peak beta weights were selected independently from the time course of each single participant. We assessed significance using t-tests on the resulting peak beta weights. All t-tests were two-sided. To control for familywise error rate the significance level was adjusted, whenever doing three or more comparisons, using the Holm-Bonferroni method.

Specify type of analysis: ☐ Whole brain ☐ ROI-based ☒ Both

Anatomical location(s)

To create anatomical regions of interest (ROI) anatomical masks were designed for each ROI in the MNI standard space using the Harvard-Oxford Subcortical Structural Atlas, Atlas of the Human Brain (Mai et al.) and atlas of human subcortical brain nuclei (Pauli et al.). Masks were then transformed from the standard space to each participant’s structural space by applying a standard-to-structural warp field and from structural to functional space by applying a structural-to-functional affine matrix. Transformed masks were thresholded (at 0.5), binarized and were dilated by one voxel. Functional ROIs (anterior insula and SMA) were defined as spheres of 1.5 mm radius, centred at the centroid of local maxima (peaks) of an activation cluster.

Statistic type for inference
(See Eklund et al. 2016)

For whole-brain analysis, the results were cluster-corrected with the voxel inclusion threshold of $Z = 3.1$ and cluster significance threshold of $P = 0.001$. The data were pre-whitened before analysis to account for temporal autocorrelations. For time-series analysis we used a leave-one-out method on group peak signals (see above).

Correction

FWE was used with $p<0.001$

## Models & analysis

| n/a | Involved in the study | |
|---|---|---|
| ☐ | ☒ | Functional and/or effective connectivity |
| ☒ | ☐ | Graph analysis |
| ☒ | ☐ | Multivariate modeling or predictive analysis |

Functional and/or effective connectivity

Psycho-physiological interaction (PPI) analyses were performed. Using the ROI procedure described above, we extracted time-series data from habenula that served as a seed region (that is the physiological regressor: PHY). Our psychological (PSY) task regressor was the 'willingness-to-act'.
To investigate how regions are connected at a wider circuit level, we conducted structural equation modelling (SEM) to probe for covariance between regions in the time-course of BOLD response. All structural equation modelling was conducted in Latent Variable Analysis (lavaan) package v.0.6–4 using Maximum Likelihood estimation. Akaike information criterion (AIC) was used for model comparison.
